# Identifications of Similarity Metrics for Patients With Cancer: Protocol for a Scoping Review

**DOI:** 10.2196/58705

**Published:** 2024-09-04

**Authors:** Iryna Manuilova, Jan Bossenz, Annemarie Bianka Weise, Dominik Boehm, Cosima Strantz, Philipp Unberath, Niklas Reimer, Patrick Metzger, Thomas Pauli, Silke D Werle, Susann Schulze, Sonja Hiemer, Arsenij Ustjanzew, Hans A Kestler, Hauke Busch, Benedikt Brors, Jan Christoph

**Affiliations:** 1 Junior Research Group (Bio-) Medical Data Science Faculty of Medicine Martin Luther University Halle-Wittenberg Halle (Saale) Germany; 2 Data Integration Centre University Hospital Halle (Saale) Halle (Saale) Germany; 3 Medical Center for Information and Communication Technology Universitätsklinikum Erlangen Friedrich-Alexander-Universität Erlangen-Nürnberg Erlangen Germany; 4 Bavarian Cancer Research Center (Bayerisches Zentrum für Krebsforschung) Erlangen Germany; 5 Medical Informatics Institute for Medical Informatics, Biometrics and Epidemiology Friedrich-Alexander-Universität Erlangen-Nürnberg Erlangen Germany; 6 SRH Fürth University of Applied Sciences Fürth Germany; 7 Medical Systems Biology Group Lübeck Institute of Experimental Dermatology University of Lübeck Lübeck Germany; 8 University Cancer Center Schleswig-Holstein University Hospital Schleswig-Holstein Lübeck Germany; 9 Medical Data Integration Center University Hospital Schleswig-Holstein Lübeck Germany; 10 Institute of Medical Bioinformatics and Systems Medicine Medical Center, Faculty of Medicine University of Freiburg Freiburg Germany; 11 Clinical Trial Office German Cancer Research Center (DKFZ) Heidelberg Heidelberg Germany; 12 Institute of Medical Systems Biology Ulm University Ulm Germany; 13 Krukenberg Cancer Center Halle (Saale) Halle (Saale) Germany; 14 Institute of Medical Biostatistics, Epidemiology and Informatics (IMBEI) University Medical Center of the Johannes Gutenberg-University Mainz Mainz Germany; 15 Division of Applied Bioinformatics German Cancer Research Center (DKFZ) Heidelberg Germany; 16 German Cancer Consortium Heidelberg Germany; 17 National Center for Tumor Diseases (NCT) Heidelberg Germany; 18 Medical Faculty Heidelberg and Faculty of Biosciences Heidelberg University Heidelberg Germany

**Keywords:** patient similarity, cancer research, patient similarity applications, precision medicine, cancer similarity metrics, scoping review protocol

## Abstract

**Background:**

Understanding the similarities of patients with cancer is essential to advancing personalized medicine, improving patient outcomes, and developing more effective and individualized treatments. It enables researchers to discover important patterns, biomarkers, and treatment strategies that can have a significant impact on cancer research and oncology. In addition, the identification of previously successfully treated patients supports oncologists in making treatment decisions for a new patient who is clinically or molecularly similar to the previous patient.

**Objective:**

The planned review aims to systematically summarize, map, and describe existing evidence to understand how patient similarity is defined and used in cancer research and clinical care.

**Methods:**

To systematically identify relevant studies and to ensure reproducibility and transparency of the review process, a comprehensive literature search will be conducted in several bibliographic databases, including Web of Science, PubMed, LIVIVIVO, and MEDLINE, covering the period from 1998 to February 2024. After the initial duplicate deletion phase, a study selection phase will be applied using Rayyan, which consists of 3 distinct steps: title and abstract screening, disagreement resolution, and full-text screening. To ensure the integrity and quality of the selection process, each of these steps is preceded by a pilot testing phase. This methodological process will culminate in the presentation of the final research results in a structured form according to the PRISMA-ScR (Preferred Reporting Items for Systematic Reviews and Meta-Analyses extension for Scoping Reviews) flowchart. The protocol has been registered in the *Journal of Medical Internet Research*.

**Results:**

This protocol outlines the methodologies used in conducting the scoping review. A search of the specified electronic databases and after removing duplicates resulted in 1183 unique records. As of March 2024, the review process has moved to the full-text evaluation phase. At this stage, data extraction will be conducted using a pretested chart template.

**Conclusions:**

The scoping review protocol, centered on these main concepts, aims to systematically map the available evidence on patient similarity among patients with cancer. By defining the types of data sources, approaches, and methods used in the field, and aligning these with the research questions, the review will provide a foundation for future research and clinical application in personalized cancer care. This protocol will guide the literature search, data extraction, and synthesis of findings to achieve the review’s objectives.

**International Registered Report Identifier (IRRID):**

DERR1-10.2196/58705

## Introduction

### Background

Rapid advances in precision medicine have revolutionized cancer research, opening new opportunities to develop an unprecedented, new, personalized view of each patient. The concept of precision medicine is seemingly simple; similar patients with similar characteristics share similar outcomes. By identifying important patient characteristics and traits, the search for similar patients contributes to the pursuit of precision medicine that may determine clinical outcomes through more precise targeting of treatment by genetic, biomarker, phenotypic, or psychosocial characteristics that differentiate a given patient from others with similar clinical presentations [1-4]. The ever-increasing volume and availability of health-related data is currently challenging the broad definitions of patient groups set out in the clinical practice guidelines. Defining a similarity measure that can handle the high-dimensional space of patient data is an essential step to enable the stratification of patients into clinically meaningful subgroups [4-6]. The complex interaction between personalized patient treatment and the application of aggregate data underlines the fundamental understanding of modern oncology, which is based on the main principle that each patient has a deeply individual nature of their illness, and each case is special [7-9]. However, there is a parallel paradigm that demonstrates the essential role of applying existing data in improving the understanding of the individuality of cancer and optimizing the approach to personalized treatments. It suggests that a deep understanding of each patient’s unique characteristics and subsequent selection of therapeutic strategies can be greatly improved by identifying similarities between patients with cancer. This approach indicates that the most effective individualized treatment strategies do not develop independently but instead result from comprehensive comparison and analysis of aggregate patient data [10,11].

Patient similarity is a topic of significant interest and research in various areas of precision medicine, including cancer research. Some studies have explored the concept of patient similarity across different dimensions, such as genomics, clinical characteristics, treatment responses, and outcomes [1,5,12,13].

Despite the extensive interest in this area, there is currently no systematic approach to clarify precisely what is understood by the concept of “patient similarity” in cancer research [6]. While individual studies may use various methodologies and metrics to assess patient similarity, there is a lack of consensus on combined approaches and definitions [4,6,9]. This creates an opportunity for further research to explore and define patient similarity more comprehensively.

In addition, the definition and evaluation of common similarity metrics in cancer research that involves careful evaluation of both quantitative and qualitative factors need to be systemized. These metrics can serve as a powerful method for furthering the understanding of cancer and improving personalized patient care [6,9]. Faced with all these research gaps, we want to conduct a scoping review.

### Aim and Research Questions

The goal of our planned research is to collect and describe the existing knowledge that could help in defining and exploring how patient similarity is determined in cancer research and care. The scoping review addresses the following research questions, outlined in Textbox 1.

**Research questions.**

**Main research question:**

- What is understood by the concept of “patient similarity” in cancer research?

**Secondary questions:**

- What types of data sources are used to identify similarities between patients with cancer?
  Molecular genetic data
  Clinical data
  Therapies or treatment
  Histological data
- What different approaches and methods are used to identify and analyze similarities between patients with cancer and what clinical relevance they have?
- Which types of cancer have been the most frequently researched when it comes to finding similarities between patients?
- What challenges and limitations have been observed in the existing literature when identifying similarities between patients with cancer?

To the best of our knowledge, no scoping review has addressed the research questions proposed by this review.

## Methods

### Overview

To ensure a transparent review process, our methodology will follow the PRISMA-ScR (Preferred Reporting Items for Systematic Reviews and Meta-Analyses extension for Scoping Reviews) checklist and the Joanna Briggs Institute Reviewer’s Manual on scoping reviews [14,15]. The methodological process of conducting a scoping review will be iterative. Given this, it is expected that there may be some deviations from the originally developed a priori protocol, as a natural part of the iterative process, to refine and improve the review as it progresses. To ensure transparency in the conduct of the review, any deviations from the original protocol will be explicitly documented and reflected in the final paper of the review.

### Main Concepts and Keywords

**Figure 1:**
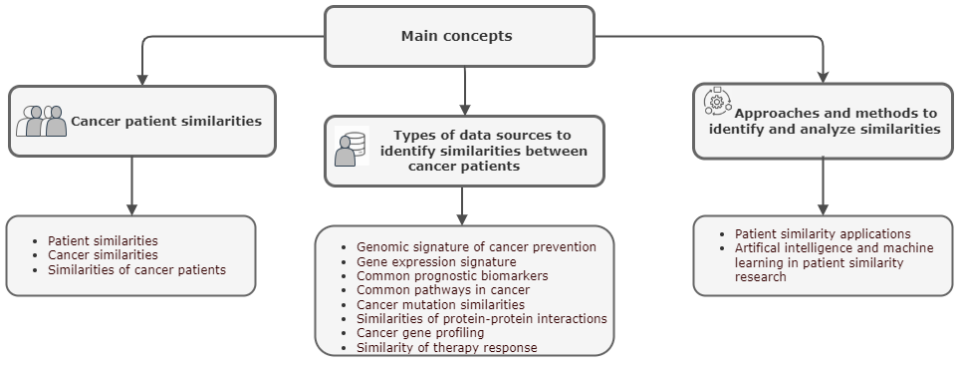
Main concepts of planned scoping review and corresponding keywords.

To guide the literature search, ensure the relevance of included studies, and improve the efficiency of the planned review process, 3 basic concepts and corresponding keywords were defined, which are graphically represented in Figure 1. Based on the main goals and research questions of this review, 3 basic concepts with corresponding keywords were selected (Figure 1), and peer review was sought from experts in the field to validate the keyword selection. These keywords guided the literature search, ensured the relevance of included studies, and improved the efficiency of the planned review process. The first concept is “Cancer patient similarities.” It focuses on understanding and summarizing the various dimensions in which patients with cancer can be similar. The main keywords are “patient similarity,” “similarities of cancer patients,” and “cancer similarity metrics.” All these terms are broad and encompass any research comparing various aspects of patients, such as molecular genetic features, clinical features, treatment outcomes, survival rates, and applications used to investigate these similarities. The second concept is “Types of data sources to identify similarities between cancer patients.” The keywords in this category expand on the first category by providing detailed perspectives that define cancer similarity metrics. These are essential for identifying studies that explore similarities from different views, such as common prognostic biomarkers, protein-protein interaction pathways, gene expression analysis, and cancer gene profiling. The third concept is “Approaches & methods to identify and analyze similarities,” with the overarching keyword being “patient similarity applications.” This category aims to identify potential methods, technologies, and algorithms that can be applied in cancer research regarding patient similarities.

### Eligibility Criteria: Inclusion and Exclusion

Even though our primary goal is to cover a wide range of studies to ensure broad coverage of studies on similarities to patients with cancer, we adhere to minimum exclusion criteria to maintain the quality and relevance of included studies. Pilot testing of eligibility criteria was performed on a sample of studies. Based on the findings, inclusion and exclusion criteria were refined to ensure robustness and effectiveness in capturing relevant studies. For selecting pertinent studies for planned scoping review, we have established the following inclusion and exclusion criteria, outlined in Textbox 2.

**Eligibility criteria.**

**Inclusion criteria**

- Type of study: All types of studies providing substantial evidence or data relevant to cancer patient similarities.
- Population: Studies focusing on patients with cancer of all ages, genders, and ethnicities with different diagnoses.
- Publications: Studies published within the last 25 years.
- Language: Studies published in English or German. (We chose English and German as the primary languages since they are the working languages of our team. This decision aims to facilitate better internal discussion and ensure the quality of results.)

**Exclusion criteria**

- Type of study:
  Publications not addressing the aspects of similarity of patients with cancer as defined in objectives.
  Bachelor’s and master’s theses, as well as unpublished papers.
- Population: Studies focusing on noncancer conditions or animal studies.
- Publications: Studies published more than 25 years ago.
- Language: Studies published in other languages.

### Types of Evidence

To identify potentially relevant studies and to ensure reproducibility and transparency of the planned review process, the following bibliographic databases were searched for literature coverage from 1998 to February 2024: Web of Science, PubMed, LIVIVO, and MEDLINE. These databases were chosen for their comprehensive coverage of the biomedical and health care literature, ensuring a thorough review of studies regarding similarities of patients with cancer over 25 years. This approach ensures reproducibility and transparency of the review, facilitating detailed analysis of existing evidence and identification of research gaps in the field [14,16].

### Search Strategy

As a result of the numerous discussions, the team developed a search strategy with 3 important steps that are keyword search, snowball system, and manual search. In the planned systematic review, keyword searching will serve as the primary method for identifying relevant studies. This approach involves the use of carefully selected keywords and keyword combinations defined using a nesting approach involving Boolean operators and field tags to provide precision (more information in Multimedia Appendix 1). Initially, the search will be conducted in the Web of Science database, and after that, the search queries will be refined and adapted for subsequent use in other chosen databases to identify relevant information on the research topic effectively. To reduce irrelevant findings in our research and to make it more exact [17,18], we integrated the Medical Subject Headings (MeSH) option (MeSH=neoplasms) with keyword searches. This was directly applied in Ovid MEDLINE and PubMed. However, we did not apply it in the Web of Science, which uses its unique indexing system [19]. To uncover literature that may have been missed after the initial keyword search, a “snowballing” method was applied [20,21]. This involved reviewing the references of the searched papers to identify additional studies not covered in the initial database search. In addition to the keywords and snowballing searches, a manual search will be conducted. This will involve manually scanning relevant journals, conference proceedings, and other literature sources to identify studies that are not indexed in mainstream databases or published in less accessible formats. Applying this triangular search strategy to the main concepts identified in Figure 1 will provide a robust review of the existing literature and will allow the fullest possible range of studies to be integrated to identify potential similarities between patients with cancer. The search options used in the individual databases are optimized to the strengths and specific functions of each platform to maximize the effectiveness and comprehensiveness of the literature selection.

### Data Extraction

Following the search, all identified references will be collected and uploaded to the reference management software package, EndNote (version 20.2.1, Clarivate Plc), where duplicates will be removed. Subsequently, we will use a selection process by our multidisciplinary team as proposed by Guar et al [22] and Schwenker et al [23], using Rayyan, a web-based software designed to facilitate the process of conducting various types of reviews [24]. The study selection process will consist of 3 stages: title-abstract screening, disagreement resolution, and finally, full-text screening, outlined in Textbox 3. To ensure the quality of the overall study selection process, pilot testing will precede each step, and the following calibrated forms will be applied. The final results will be represented using the PRISMA-ScR flowchart [14].

**Stages of the study selection process.**

**Stage 1: Title-abstract screening**

In this first step, we will screen titles and abstracts to quickly filter out publications that are not relevant to our research questions. This step will significantly reduce the volume of work required in the subsequent full-text review phase. To ensure objectivity, at least 2 reviewers in blind mode will screen each paper. A total of 11 reviewers participated in the title-abstract screening process.

**Stage 2: Disagreement resolution**

Discrepancies during data extraction will be resolved based on the decision of an additional reviewer, as suggested by von Elm et al [15]. Each reviewer will extract data independently. In case of disagreements, the independent reviewer will be consulted to make the final decision. All discrepancies and their resolutions will be documented for transparency.

**Stage 3: Full-text screening**

After the primary selection, we will conduct a full-text review of the remaining papers to further refine our selection based on specific inclusion and exclusion criteria directly related to our research questions. A total of 9 independent reviewers will be involved at this stage, as well as in the data extraction process.

### Management of Data Charting

From all publications that will be included in the research after the full-text screening stage, data will be extracted. The data extraction process is manual, performed by our team using a predefined template to ensure systematic and accurate data capture. By using a predefined data extraction template, we can systematically capture all relevant information from each study, maintaining consistency and minimizing errors. Pilot testing of the template was conducted to ensure robustness and accuracy. The final draft extraction form is provided in Table 1.

This template is designed with several sections to capture essential information from the studies; “Metadata” includes general information about the publication, and “Research findings” summarizes the main findings from each paper, specific to the research questions and objectives of the planned scoping review. The process of data charting, as in the case of the selection of sources of evidence, will start with a calibration step, which will help us prevent errors and ensure high interrater agreement [14].

**Table 1:** Data extraction table for the scoping review.

Item				Description		Keypoints
**Metadata^a^**						
	Title			Title		—^b^
	Details			Author (first), journal, DOI^c^		—
	Year of publication			YYYY		—
	Publication type			Type of publication		—
	Institute			Corresponding institute		—
	Objective			The main objective of the publication		—
	Methods			Summary of the proposed methodological approach		—
	Results			Short description of the results		—
	Conclusion			Summarizing the main points and findings		—
	Keywords			Main keywords of the publication		—
**Research findings**						
	**Main research question**					
		What is understood by the concept of “patient similarity” in cancer research?	Key definition of “patient similarity” in the context of the publication.		Explanation of how the study defines patient similarity in the context of cancer research. This can include genetic, clinical, histological treatment-related similarities, or view from methodological approaches. Determining the aspects of patient similarity that this publication focused on.	
	**Secondary questions**					
		What types of patient data are used to identify similarities?	Short description of the data (molecular genetics, clinical, histologic, and treatment-related) used to define patient similarity.		Categorization of the types of patient data used to identify similarities.	
		What different approaches and methods are used to identify and analyze similarities between patients with cancer and what clinical relevance they have?	The approaches and methods used to analyze and identify similarities (eg, software, tools, and algorithms). Information on how these findings contribute to personalized medicine.		Typification of the tools used to identify similarities. Clinical relevance of the methods and suitability for practical application.	
		Which types of cancer have been the most frequently researched when it comes to finding similarities between patients?	A list of cancer types that can be related as a basis for identifying similar cancer metrics.		Identification of cancer types associated with the patient similarities in this study.	
		What challenges and limitations have been observed in the existing literature when identifying similarities between patients with cancer?	List of potential limitations and challenges.		Determination of the limits, future challenges, and unexplored areas in this field of research.	

^a^Mandatory field.

^b^Not applicable.

^c^DOI: digital object identifier.

### Summarizing and Presenting Results

To comprehensively answer the main research question and related secondary questions, our findings will be summarized and presented using a structured approach to ensure clarity, consistency, and alignment with overarching objectives. Detailed narrative synthesis and descriptive analysis will provide the basis for summarizing and presenting the findings of the studies [14,24,25], included in the review, focusing on how “patient similarity” is conceptualized and operationalized within cancer research. This process will summarize key findings and thematic categories and establish links between approaches to “cancer patient similarity” across studies. Additional graphical and tabular forms will be used to visualize and systematically present the collected data. For this purpose, we are planning to include flowcharts representing the study selection process, diagrams, and bar charts illustrating the intersection of different types of data sources or showing the frequency of studies of different cancer types.

### Ethical Considerations

There was no requirement for ethical approval because only literature was being evaluated.

## Results

The review protocol, which outlines the methodology for the review, began with a database search, identifying 1183 unique papers after the removal of duplicates. As the review advanced to full-text screening by March 2024, the selection process led to 67% (797/1183) of the papers being excluded and 13% (151/1183) of papers being earmarked for conflict resolution. Consequently, 20% (235/1183) of selected papers, which significantly contribute to the analysis of the review and align with the research questions and objectives, were initially considered for inclusion. This number rose to 22% (258/1183) after resolving conflicts. Currently, a full-text analysis is underway using a pretested chart template to ensure that each selected study contributes to the comprehensive understanding the review aims to establish.

## Discussion

Our planned scoping review will offer insights into how the concept of patient similarity in patients with cancer has been defined and interpreted thus far. In addition, the review aims to provide an overview of the methods used to identify similarities and differences among patients with cancer. It will also specify the types of data used in these methods. Furthermore, it will provide an overview of the types of cancer addressed in the studies we cover. However, the scoping review of similarity measures for patients with cancer may face limitations, including the possibility of missing specific study details due to its broad coverage, variability in study design, diversity of data sources, and possible publication bias. In addition, rapid advances in the field and subjectivity in study selection may affect the comprehensiveness and accuracy of the review. Despite these limitations, it is important to note that the advantages and benefits of conducting such studies far outweigh the possible disadvantages, offering valuable insights into personalized cancer treatment strategies. First, it facilitates a more nuanced understanding of cancer’s biological diversity, recognizing that while each case is unique, there are often underlying similarities that can guide treatment [5]. In addition, the benefits of studying patient similarities also include the potential for more effective and targeted therapies, improving prognostic models, and discovering new approaches. Finally, identifying indicators of similarity supports ongoing treatment by allowing one to act more efficiently and effectively, armed with knowledge drawn from a broader data set [11,26]. Our review will examine the scope, range, and nature of similarity studies of patients with cancer. It will highlight key findings, identify research gaps, and explore new methods for assessing patient similarity. It will also suggest future directions for research. These directions may include patient-centered approaches by incorporating patient-reported outcomes and experiences into the definition of similarity measures. Thereby, we aim to ensure the relevance and applicability of findings in clinical practice. This is of special importance when the focus is on rare cancers, which are often underrepresented in studies. To understand if and how similarities can be identified and used in these cases, we will also consider longitudinal studies exploring patient similarities and their impact on treatment outcomes.

## Appendix

Multimedia Appendix 1 Proposed search queries for Web of Science, PubMed, LIVIVO, and MEDLINE.

## Abbreviations

MeSH: Medical Subject Headings
PRISMA-ScR: Preferred Reporting Items for Systematic Reviews and Meta-Analyses extension for Scoping Reviews

## References

[1] Dai L, Zhu H, Liu D Patient similarity: methods and applications. arXiv.

[2] Brown SA, Chung BY, Doshi K, Hamid A, Pederson E, Maddula R, Hanna A, Choudhuri I, Sparapani R, Bagheri Mohamadi Pour M, Zhang J, Kothari AN, Collier P, Caraballo P, Noseworthy P, Arruda-Olson A (2023). Patient similarity and other artificial intelligence machine learning algorithms in clinical decision aid for shared decision-making in the Prevention of Cardiovascular Toxicity (PACT): a feasibility trial design. Cardiooncology.

[3] Brown SA (2016). Patient similarity: emerging concepts in systems and precision medicine. Front Physiol.

[4] Murciano-Goroff YR, Suehnholz SP, Drilon A, Chakravarty D (2023). Precision oncology: 2023 in review. Cancer Discov.

[5] Parimbelli E, Marini S, Sacchi L, Bellazzi R (2018). Patient similarity for precision medicine: a systematic review. J Biomed Inform.

[6] Li H, Zhou M, Sun Y, Yang J, Zeng X, Qiu Y, Xia Y, Zheng Z, Yu J, Feng Y, Shi Z, Huang T, Tan L, Lin R, Li J, Fan X, Ye J, Duan H, Shi S, Shu Q (2024). A patient similarity network (CHDmap) to predict outcomes after congenital heart surgery: development and validation study. JMIR Med Inform.

[7] Corti C, Cobanaj M, Dee EC, Criscitiello C, Tolaney SM, Celi LA, Curigliano G (2023). Artificial intelligence in cancer research and precision medicine: applications, limitations and priorities to drive transformation in the delivery of equitable and unbiased care. Cancer Treat Rev.

[8] Mateo J, Steuten L, Aftimos P, André Fabrice, Davies M, Garralda E, Geissler J, Husereau D, Martinez-Lopez I, Normanno N, Reis-Filho JS, Stefani S, Thomas DM, Westphalen CB, Voest E (2022). Delivering precision oncology to patients with cancer. Nat Med.

[9] Fang HSA, Tan NC, Tan WY, Oei RW, Lee ML, Hsu W (2021). Patient similarity analytics for explainable clinical risk prediction. BMC Med Inform Decis Mak.

[10] Victoir B, Croix C, Gouilleux F, Prié Gildas (2024). Targeted therapeutic strategies for the treatment of cancer. Cancers (Basel).

[11] Xu J, Yang P, Xue S, Sharma B, Sanchez-Martin M, Wang F, Beaty KA, Dehan E, Parikh B (2019). Translating cancer genomics into precision medicine with artificial intelligence: applications, challenges and future perspectives. Hum Genet.

[12] Boniolo G, Campaner R, Carrara M (2021). Patient similarity in the era of precision medicine: a philosophical analysis. Erkenn.

[13] Sun J, Fei Wang (2015). PSF: a unified patient similarity evaluation framework through metric learning with weak supervision. IEEE J Biomed Health Inform.

[14] Tricco AC, Lillie E, Zarin W, O'Brien KK, Colquhoun H, Levac D, Moher D, Peters MDJ, Horsley T, Weeks L, Hempel S, Akl EA, Chang C, McGowan J, Stewart L, Hartling L, Aldcroft A, Wilson MG, Garritty C, Lewin S, Godfrey CM, Macdonald MT, Langlois EV, Soares-Weiser K, Moriarty J, Clifford T, Tunçalp Özge, Straus SE (2018). PRISMA Extension for Scoping Reviews (PRISMA-ScR): checklist and explanation. Ann Intern Med.

[15] von Elm E, Schreiber G, Haupt CC (2019). Methodische anleitung für scoping reviews (JBI-methodologie) [Article in German]. Z Evid Fortbild Qual Gesundhwes.

[16] Bramer WM, Rethlefsen ML, Kleijnen J, Franco OH (2017). Optimal database combinations for literature searches in systematic reviews: a prospective exploratory study. Syst Rev.

[17] Baumann N (2016). How to use the medical subject headings (MeSH). Int J Clin Pract.

[18] Richter RR, Austin TM (2012). Using MeSH (medical subject headings) to enhance PubMed search strategies for evidence-based practice in physical therapy. Phys Ther.

[19] Burns CS, Shapiro RM, Nix T, Huber JT (2019). Search results outliers among MEDLINE platforms. J Med Libr Assoc.

[20] Badampudi D, Wohlin C, Petersen K (2015). Experiences from using snowballing and database searches in systematic literature studies.

[21] Rønn Camille, Wieland A, Lehrer C, Márton Attila, LaRoche J, Specker A, Leroy P, Fürstenau Daniel (2023). Circular business model for digital health solutions: protocol for a scoping review. JMIR Res Protoc.

[22] Gaur A, Kumar M (2018). A systematic approach to conducting review studies: An assessment of content analysis in 25 years of IB research. J World Business.

[23] Schwenker R, Kroeber Eric Sven, Deutsch Tobias, Frese T, Unverzagt Susanne (2021). Identifying patients with psychosocial problems in general practice: a scoping review protocol. BMJ Open.

[24] Ouzzani M, Hammady H, Fedorowicz Z, Elmagarmid A (2016). Rayyan-a web and mobile app for systematic reviews. Syst Rev.

[25] Khalil H, Ameen D, Zarnegar A (2022). Tools to support the automation of systematic reviews: a scoping review. J Clin Epidemiol.

[26] Kim S, Herazo-Maya JD, Kang DD, Juan-Guardela BM, Tedrow J, Martinez FJ, Sciurba FC, Tseng GC, Kaminski N (2015). Integrative phenotyping framework (iPF): integrative clustering of multiple omics data identifies novel lung disease subphenotypes. BMC Genomics.

