# Supplementary material for: Identifications of Similarity Metrics for Patients With Cancer: Protocol for a Scoping Review
Source: JMIR Res Protoc. 2024 Sep 4;13:e58705. doi: 10.2196/58705 (PMC11411229; doi:10.2196/58705)
Supplement: Multimedia Appendix 1 [file resprot_v13i1e58705_app1.docx]

|  | | | | |
| --- | --- | --- | --- | --- |
| **MAIN CONCEPTS** | **BIBLIOGRAPHIC DATABASES** | | | |
|  | **World of Science** | **LIVIVO** | **Ovid MEDLINE** | **PubMed** |
| Cancer patient similarities | (TS=cancer OR TS=tumor) AND (TS="patient* similarit*" OR TI="similar* patient*" OR TS ="similar patient group*") | (cancer OR tumor OR MESH=neoplasms) AND ("patient similarity" OR "patients similarity" OR "patient similarities" OR "patients similarities"OR TITLE="similar patient" OR TITLE="similar patients" OR "similar patient group" OR "similar patient groups") | (cancer OR tumor OR neoplasms.sh.) AND („patient* similarit*" OR "similar* patient*".ti. OR "similar patient group*") | (cancer[Text Word] OR tumor[Text Word] OR Neoplasms[Mesh Terms]) AND ("patient similarit*"[Text Word] OR "patients similarit*"[Text Word] OR "similar patient*"[Title] OR "similarly patient*"[Title] or "similar patient group*"[Text Word]) |
|  | TS=(("precision medicine" OR "personalized medicine") AND (cancer OR tumor) AND patient AND similarit*) | ("precision medicine" OR "personalized medicine") AND (cancer OR tumor OR MESH=neoplasms) AND patient AND similarit* | ("precision medicine" OR "personalized medicine") AND (cancer OR tumor OR neoplasms.sh.) AND patient AND similarit* | ("precision medicine"[Text Word] OR "personalized medicine"[Text Word]) AND (cancer[Text Word] OR tumor[Text Word] OR Neoplasms[Mesh Terms]) AND patient[Text Word] AND similarit*[Text Word] |
|  | TS=((("genomic similarit*") OR ("similarit* genomics") OR ("omic similarit*") OR ("similarit* omics"))AND (cancer OR tumor)) | ("genomic similarities" OR "genomic similarity" OR "similarity genomics" OR "similarities genomics" OR "omic similarity" OR "omic similarities" OR "similarity omics") AND (cancer OR tumor OR MESH=neoplasms) | (("genomic similarit*") OR ("similarit* genomics") OR ("omic similarit*") OR ("similarit* omics"))AND (cancer OR tumor OR neoplasms.sh.) | (("genomic similarit*"[Text Word]) OR ("similarity genomics"[Text Word]) OR ("omic similarit*"[Text Word]) OR ("similarity omics"[Text Word])) AND (cancer[Text Word] OR tumor[Text Word] OR Neoplasms[Mesh Terms]) |
| Types of data sources | TS=((("gene expression signature*") OR ("genomic signature*")) AND (cancer OR tumor) AND patient AND similarit*) | ("gene expression signature" OR "gene expression signatures" OR "genomic signature" OR "genomic signatures") AND (cancer OR tumor) AND patient AND similarit* | (("gene expression signature*") OR ("genomic signature*")) AND (cancer OR tumor OR neoplasms.sh.) AND patient AND similarit* | (("gene expression signature*"[Text Word]) OR ("genomic signature*"[Text Word])) AND (cancer[Text Word] OR tumor[Text Word] OR Neoplasms[Mesh Terms]) AND patient[Text Word] AND similarit*[Text Word] |
|  | TS=((biomarker) AND (cancer OR tumor) AND patient AND similarit*) | "biomarker*" AND (cancer OR tumor OR MESH=neoplasms) AND patient AND similarit* | (biomarker) AND (cancer OR tumor OR neoplasms.sh.) AND patient AND similarit* | (biomarker[Text Word]) AND (cancer[Text Word] OR tumor[Text Word] OR Neoplasms[Mesh Terms]) AND patient[Text Word] AND similarit*[Text Word] |
| Types of data sources | TS=(("common pathway*" OR "similar pathway*") AND (cancer OR tumor) AND patient AND similarit*) | ("common pathway" OR "common pathways" OR "similar pathways" OR "similar pathway") AND (cancer OR tumor OR MESH=neoplasms) AND patient AND similarit* | ("common pathway*" OR "similar pathway*") AND (cancer OR tumor OR neoplasms.sh.) AND patient AND similarit* | ("common pathway*"[Text Word] OR "similar pathway*"[Text Word]) AND (cancer[Text Word] OR tumor[Text Word] OR Neoplasms[Mesh Terms]) AND patient[Text Word] AND similarit*[Text Word] |
|  | TS=(Similarit* AND (mutations OR "protein-protein interaction*" OR "molecular profil*") AND "cancer patient*") | Similarit* AND (mutations OR "protein-protein interaction" OR "protein-protein interactions" OR "molecular profil" OR "molecular profils" OR "molecular profiling") AND ("cancer patient" OR "cancer patients") | Similarit* AND (mutations OR "protein-protein interaction*" OR "molecular profil*") AND "cancer patient*" | Similarit*[Text Word] AND (mutations[Text Word] OR "protein protein interaction*"[Text Word] OR "molecular profil*"[Text Word]) AND "cancer patient*"[Text Word] |
|  | TS=(("therap* respons*" OR "drug respons*") AND Similarit* AND (cancer OR tumor) AND patient*) | ("therapy response" OR "therapy responses" OR "drug response" OR "drug responses") AND Similarit* AND (cancer OR tumor OR MESH=neoplasms) AND patient* | ("therap* respons*" OR "drug respons*") AND Similarit* AND (cancer OR tumor OR neoplasms.sh.) AND patient*t* | ("therapy respons*"[Text Word] OR "drug respons*"[Text Word]) AND Similarit*[Text Word] AND (cancer[Text Word] OR tumor[Text Word] OR Neoplasms[Mesh Terms]) AND patient*[Text Word] |
| Approaches & Methods | TS=("patient similarit*" AND ("Network*" OR "method*" OR "application*" OR "model*" OR "analysis*") AND (cancer OR tumor)) | ("patient similarity" OR "patient similarities")AND ("Network*" OR "method*" OR "application*" OR "model*" OR "analysis*") AND(cancer OR tumor OR MESH=neoplasms) | "patient similarit*" AND ("Network*" OR"method*" OR "application*" OR "model*" OR "analysis*") AND (cancer OR tumor OR neoplasms.sh.) | "patient similarit*"[Text Word] AND ("Network*"[Text Word] OR "method*"[Text Word] OR "application*"[Text Word] OR "model*"[Text Word] OR "analysis*"[Text Word]) AND (cancer[Text Word] OR tumor[Text Word] OR Neoplasms[Mesh Terms]) |
|  | TS=( "patient similarit*" AND ("Machine learning*" OR "Clustering*" OR "Artificial intelligence" OR "graph network*" OR "deep learning")) | ("patient similarity" OR "patient similarities") AND ("Machine learning*" OR "Clustering*" OR "Artificial intelligence" OR "graph network" OR "graph networks" OR "deep learning") | "patient similarit*" AND ("Machine learning*" OR "Clustering*" OR "Artificial intelligence" OR "graph network*" OR "deep learning") | "patient similarit*"[Text Word] AND ("Machine learning*"[Text Word] OR "Clustering*"[Text Word] OR "Artificial intelligence"[Text Word] OR "graph network*"[Text Word] OR "deep learning"[Text Word]) |
